# Supplementary material for: Tree height-diameter allometry and implications for biomass estimates in Northeastern Amazonian forests
Source: PeerJ. 2025 Mar 11;13:e18974. doi: 10.7717/peerj.18974 (PMC11908443; doi:10.7717/peerj.18974)
Supplement: Supplemental Information 1 — Provide descriptions about the analytical methods used for extraction of the phylogenetic tree, phylogenetic signals and for regression using phylogenetic generalized least squares. [file peerj-13-18974-s001.doc]

**Appendix S1: Description of phylogenetic autocorrelation analysis.**

We extracted a phylogenetic tree for 417 taxa (88.25% identified up to species, 11.51% up to genus and 0.24% up to family level) from the supertree proposed by Smith & Brown (2018) for seed plants. Genera and species were added to their families or genera using simple phylogenetic branch length adjuster (BLADJ) (*Webb, 2000*). Using the extracted phylo-tree we calculated the phylogenetic signals for maximum height and maximum diameter at breast height (DBH) with Pagel's lambda (*Pagel, [1999](https://nph.onlinelibrary.wiley.com/doi/10.1111/nph.14123#nph14123-bib-0037)*). We chose the maximum values of height and DBH for each taxon to minimize the variation associated to tree age.

We used phylogenetic generalized least squares (PGLS) to test for the relationship between maximum height and maximum DBH for terra-firme, várzea and both forests. This technique uses the phylogenetic relationships between taxa to generate regression estimates that account for the interspecific correlation due to the phylogeny (*Symonds & Blomberg, 2014*). We used the maximum likelihood as estimator and a covariance matrix expected under Brownian model (Martins & Hansen, 1997). We used the best height-diameter model chosen in the selection procedure, and applied PGLS to estimate the parameters of the relationship between maximum height (*H*_max_) and maximum DBH (*D_max_*): *H_max_ = a + b × ln(D_max_) + c × ln(D_max_)*^2^ *+ Σ*, where *Σ* is the phylogenetic covariance matrix calculated on branch lengths of the phylogenetic tree. Further, we compared the performance of this model (using RSE, adjusted R^2^ and AIC) to the model run with least square regression without accounting for phylogenetic structure.

The extraction of the phylo-tree was done using the *V.PhyloMaker* package (*Jin & Qian, 2019*). Phylogenetic signals for traits were calculated using the *phytools* package, version 1.0 (*Revell, 2012*) and PGLS model was run in the *nlme* package, version 3.1 (*Pinheiro et al., 202*2) and the covariance matrix was calculated in the *ape* package, version 5.6 (*Paradis & Schliep, 2019*).

REFERENCES

**Jin Y, Qian H. 2019**. V.PhyloMaker: an R package that can generate very large phylogenies for vascular plants. Ecography **42**:1353–1359 DOI 10.1111/ecog.04434

**Martins EP, Hansen TF. 1997**. Phylogenies and the comparative method: a general approach to incorporating phylogenetic information into the analysis of interspecific data. American Naturalist **149**:646–667.

**Pagel M. 1999**. Inferring the historical patterns of biological evolution. Nature **401**:877–884 DOI 10.1038/44766

**Paradis E, Schliep K. 2019**. ape 5.0: an environment for modern phylogenetics and evolutionary analyses in R. Bioinformatics **35**:526–528.

**Pinheiro J, Bates D, R Core Team. 2022**. nlme: Linear and Nonlinear Mixed Effects Models. R package version 3.1-157. <https://CRAN.R-project.org/package=nlme>.

**Revell LJ. 2012.** phytools: an R package for phylogenetic comparative biology (and other things). Methods in Ecology and Evolution **3**:217–223. DOI 10.1111/j.2041-210x.2011.00169.x

**Smith SA, Brown JW. 2018**. Constructing a broadly inclusive seed plant phylogeny. American Journal of Botany **105**:302-314. DOI 10.1002/ajb2.1019

**Symonds MRE, Blomberg SP. 2014**. A primer on phylogenetic generalised least squares. In: Garamszegi LZ, ed. Modern phylogenetic comparative methods and their application in evolutionary biology: concepts and practice. Berlin Heidelberg: Springer, 105–130. DOI 10.1007/978-3-662-43550-2_5

**Webb CO. 2000**. Exploring the Phylogenetic Structure of Ecological Communities: An Example for Rain Forest Trees. The American Naturalist 156(2):145–155. DOI 10.1086/303378
