# Supplementary material for: Tree height-diameter allometry and implications for biomass estimates in Northeastern Amazonian forests
Source: PeerJ. 2025 Mar 11;13:e18974. doi: 10.7717/peerj.18974 (PMC11908443; doi:10.7717/peerj.18974)
Supplement: Supplemental Information 3 — The the values of Graybill’s test, model lack-of-fit, no-bias (intercept-a = 0), consistency (slope-b =1) and regression lack-of-fit for the Quadratic, Michaelis-Menten and Weibull models are followed by probabilities associated to F-test. [file peerj-13-18974-s003.pdf]

Model	Graybill´s test	*p*	Model lack-of-fit	*p*	No bias ($a = 0$)	*p*	Consistency ($b = 1$)	*p*	Regression lack-of-fit	*p*
**Terra-firme**										
Quadratic	80.0128	0	10422.4714	0.06549	0.23662	0.9036	0.63004	0.84341	10421.60474	0.06076
Michaelis-Menten	74.0156	0	10468.0004	0.0591	2.54262	0.69173	6.67975	0.52058	10458.77803	0.05581
Weibull	92.6548	0	10543.06827	0.04971	2.11507	0.71816	5.7755	0.55106	10535.17769	0.04668
**Várzea**										
Quadratic	220.1584	0	10801.73809	0	2E-05	0.99918	0.00015	0.99768	10801.73793	0
Michaelis-Menten	211.136	0	10841.29159	0	0.21176	0.91199	1.65156	0.75772	10839.42827	0
Weibull	222.844	0	10811.81963	0	0.01309	0.97806	0.10431	0.93814	10811.70223	0
**Both forests**										
Quadratic	310.9531	0	36302.50768	0	2.5345	0.74702	9.64666	0.52922	36290.32652	0
Michaelis-Menten	312.3361	0	36284.50935	0	2.16781	0.76541	8.26443	0.56024	36274.0771	0
Weibull	335.6643	0	36255.14888	0	0.08	0.95428	0.31198	0.90987	36254.7569	0
