# Supplementary material for: Tree height-diameter allometry and implications for biomass estimates in Northeastern Amazonian forests
Source: PeerJ. 2025 Mar 11;13:e18974. doi: 10.7717/peerj.18974 (PMC11908443; doi:10.7717/peerj.18974)
Supplement: Supplemental Information 4 — The RSEresidual is the residual standard error of fixed and random factor together and the RSEespecies is the error associated to species identity; the R2marginal is the variation explained by fixed the factor and the R2conditional represents the variation explained by both fixed and the random factors; and AIC is the Akaike Information Criterion. [file peerj-13-18974-s004.pdf]

**Model name**	**Parameter**	**Estimate**	***SE***	***RSE*** **species**	***RSE*** ***residual***	R^2 **marginal**	R^2 **conditional**	***AIC***
				**Terra-firme**				
Quadratic	*a*	2.10889	0.374	1.058	3.896	0.87	0.88	6496.5
	b	1.64939	0.39					
	c	1.68951	0.092					
Log-linear	*a*	-2.32125	0.33	1.7196	4.317	0.83	0.85	6788.1
	b	8.50961	0.123					
				**Várzea**				
Quadratic	*a*	-7.71048	2.184	1.652	3.916	0.64	0.69	4535.8
	b	9.05609	1.534					
	c	-0.25328	0.259					
Log-linear	*a*	-5.66142	0.628	1.647	3.916	0.64	0.69	4535.9
	b	7.56482	0.194					
				**Both forests**				
Quadratic	*a*	0.86263	0.436	2.910	4.026	0.75	0.83	11407.6
	b	4.33485	0.37					
	c	0.80812	0.078					
Log-linear	*a*	-2.1753	0.332	3.228	4.089	0.73	0.83	11506.7
	b	7.99649	0.11					
