# Supplementary material for: Tree height-diameter allometry and implications for biomass estimates in Northeastern Amazonian forests
Source: PeerJ. 2025 Mar 11;13:e18974. doi: 10.7717/peerj.18974 (PMC11908443; doi:10.7717/peerj.18974)
Supplement: Supplemental Information 5 — A quadratic model relating maximum height with maximum diameter was applied to compare PGLS with the generalized least square (GLS) regression, using RSE, Adjusted pseudo R2 and AIC. [file peerj-13-18974-s005.pdf]

Regression type	Parameter	Estimate	*SE*	*RSE*	Adj. pseudo R^2	*AIC*
			**Terra-firme**			
GLS	*a*	2.03024	0.9	4.585	0.86	2117.1
	b	1.4098	823			
	c	1.83829	174			
PGLS	*a*	1.16502	5.849	19.594	0.85	2484.1
	b	4.93838	638			
	c	0.79186	107			
			**Várzea**			
GLS	*a*	-17.4028	8.747	4.776	0.67	386.7
	b	15.14652	5.497			
	c	-1.01665	831			
PGLS	*a*	-23.77751	8.553	7.84	0.66	407.1
	b	19.29762	5.18			
	c	-1.71952	787			
			**Both forests**			
GLS	*a*	0.62485	1.042	5.426	0.79	2598.8
	b	3.77679	901			

	c	1.13152	183			
PGLS	*a*	0.37904	7.663	26.329	0.78	3138.4
	b	-0.43571	647			
	c	2.46354	155			
