# Supplementary material for: Tree height-diameter allometry and implications for biomass estimates in Northeastern Amazonian forests
Source: PeerJ. 2025 Mar 11;13:e18974. doi: 10.7717/peerj.18974 (PMC11908443; doi:10.7717/peerj.18974)
Supplement: Supplemental Information 6 — Fitted height was compared to actual height at tree level. [file peerj-13-18974-s006.pdf]

DBH class (cm)	Model	*TE* (m)	*CV TE* (%)	*SE* (m)	*CV SE* (%)	*RE* (m)	*CV RE* (%)
			**Terra-firme**				
≥1 - <30	Quadratic	3.4	25.4	0	0	3.4	25.4
	Michaelis-Menten	3.4	25.4	0	-0.2	3.4	25.4
	Weibull	3.4	25.5	0	0.3	3.4	25.5
	3 par. Exponential	3.4	25.7	0	-0.2	3.4	25.7
	2 par. Exponential	3.4	25.8	-0.3	-1.9	3.4	25.8
	Guiana Shield	3.5	26.4	-0.7	-5.5	3.4	25.8
	Pantropical	4.8	36	-2.6	-19.7	4	30.2
≥30	Quadratic	5.8	17.9	-0.1	-0.2	5.8	17.9
	Michaelis-Menten	5.8	17.9	-0.1	-0.3	5.8	18
	Weibull	5.8	17.9	0.1	0.2	5.8	17.9
	3 par. Exponential	5.9	18.1	0.1	0.4	5.9	18.1
	2 par. Exponential	5.9	18.2	0	0.1	5.9	18.3
	Guiana Shield	5.8	17.9	0.5	1.7	5.8	17.9
	Pantropical	8.4	25.9	-6.1	-18.7	5.8	18
≥1 (all trees)	Quadratic	4	23.2	0	-0.1	4	23.2
	Michaelis-Menten	4	23.2	0	-0.3	4	23.2
	Weibull	4	23.2	0	0.2	4	23.2
	3 par. Exponential	4.1	23.4	0	0	4.1	23.4
	2 par. Exponential	4.1	23.6	-0.2	-1.1	4.1	23.6
	Guiana Shield	4.1	23.7	-0.5	-2.8	4.1	23.5

	Pantropical	5.7	33.2	-3.3	-19.3	4.7	27
	**Várzea**						
≥5 - <30	Quadratic	3.6	27.2	0	0	3.6	27.3
	Michaelis-Menten	3.6	27.3	0	-0.1	3.6	27.3
	Weibull	3.6	27.3	0	-0.1	3.6	27.3
	3 par. Exponential	3.6	27.4	0	-0.4	3.6	27.4
	Log-linear	3.6	27.2	0	0.3	3.6	27.2
	Guiana Shield	5.1	38.3	2.8	21.4	4.2	31.8
	Pantropical	3.7	27.5	0.1	0.5	3.7	27.5
≥30	Quadratic	5.4	22.9	-0.1	-0.3	5.4	23
	Michaelis-Menten	5.4	23	-0.1	-0.4	5.5	23
	Weibull	5.4	22.9	0	0	5.4	23
	3 par. Exponential	5.4	23	0.1	0.4	5.4	23
	Log-linear	5.4	22.9	-0.2	-0.8	5.4	23
	Guiana Shield	11.4	48.2	10	42.2	5.5	23.2
	Pantropical	6.6	28.1	3.5	14.6	5.7	24.1
≥5 (all trees)	Quadratic	4.2	26.3	0	0	4.2	26.3
	Michaelis-Menten	4.2	26.3	0	-0.1	4.2	26.3
	Weibull	4.2	26.3	0	0	4.2	26.3
	3 par. Exponential	4.2	26.3	0	0	4.2	26.4
	Log-linear	4.2	26.3	0	0	4.2	26.3
	Guiana Shield	7.2	45.6	4.6	29.2	5.5	35.1
	Pantropical	4.7	29.5	0.9	5.9	4.6	29
	**Both forests**						
≥1 - <30	Quadratic	4	29.7	0	0.1	4	29.7

	Michaelis-Menten	4	29.7	0	0.1	4	29.7
	Weibull	4	29.8	0	0.3	4	29.8
	3 par. Exponential	4	29.9	0	0.1	4	29.9
	2 par. Exponential	4	30	-0.2	-1.2	4	30
	Guiana Shield	4.2	31.7	0.7	5.2	4.2	31.2
	Pantropical	4.4	32.9	-1.6	-11.7	4.1	30.8
≥30	Quadratic	7.3	25.7	-0.3	-0.9	7.3	25.7
	Michaelis-Menten	7.3	25.7	-0.3	-0.9	7.3	25.7
	Weibull	7.3	25.7	-0.1	-0.5	7.3	25.7
	3 par. Exponential	7.3	25.7	-0.1	-0.3	7.4	25.8
	2 par. Exponential	7.4	25.9	-0.2	-0.6	7.4	25.9
	Guiana Shield	8.8	30.8	4.8	16.8	7.4	25.8
	Pantropical	7.7	26.9	-1.8	-6.3	7.5	26.2
≥1 (all trees)	Quadratic	4.9	29.5	0	-0.2	4.9	29.5
	Michaelis-Menten	4.9	29.5	0	-0.2	4.9	29.5
	Weibull	4.9	29.5	0	0	4.9	29.5
	3 par. Exponential	4.9	29.6	0	0	4.9	29.6
	2 par. Exponential	5	29.7	-0.2	-1	5	29.7
	Guiana Shield	5.6	33.5	1.6	9.7	5.4	32.1
	Pantropical	5.3	31.9	-1.6	-9.5	5.1	30.5
