# Supplementary material for: Tree height-diameter allometry and implications for biomass estimates in Northeastern Amazonian forests
Source: PeerJ. 2025 Mar 11;13:e18974. doi: 10.7717/peerj.18974 (PMC11908443; doi:10.7717/peerj.18974)
Supplement: Supplemental Information 7 — Biomass estimates using predicted height were compared to biomass estimates using actual height at tree level. [file peerj-13-18974-s007.pdf]

**Forest type**	**DBH class (cm)**	**Best to worst model**	**Model**	***TE* (kg)**	***CV TE* (%)**	***SE* (kg)**	***CV SE* (%)**	***RE* (kg)**	***CV RE* (%)**
Terra-firme	≥1 - <30	1	Quadratic	44	35.9	1.7	1.4	43.9	35.9
		2	Michaelis-Menten	44.4	36.2	2.4	2	44.3	36.2
		3	Weibull	44.2	36.2	1.5	1.2	44.2	36.1
		4	3 par. Exponential	45	36.7	2.1	1.7	44.9	36.7
		5	2 par. Exponential	45.5	37.2	3.2	2.6	45.4	37.1
		-	Guiana Shield	44.5	36.4	-1.2	-1	44.5	36.4
		-	Pantropical	58.1	47.4	-24.9	-20.4	52.5	42.9
		-	Without height	48	39.2	-14.4	-11.8	45.8	37.4
	≥30	1	Quadratic	484.7	21.5	-4.1	-0.2	485.7	21.5
		2	Michaelis-Menten	480.3	21.3	-17.1	-0.8	481	21.3
		3	Weibull	477	21.1	-7.3	-0.3	477.9	21.1
		4	3 par. Exponential	478.6	21.2	-23.4	-1	479	21.2
		5	2 par. Exponential	486.2	21.5	-39.5	-1.7	485.6	21.5
		-	Guiana Shield	478	21.2	30.2	1.3	478.1	21.2
		-	Pantropical	628.1	27.8	-361.9	-16	514.4	22.8
		-	Without height	579.7	25.7	-32.3	-1.4	580	25.7
	≥1 (all trees)	1	Quadratic	223.9	38.9	0.6	0.1	224	38.9
		2	Michaelis-Menten	222.1	38.6	-1.9	-0.3	222.2	38.6

		3	Weibull	220.8	38.4	-0.7	-0.1	220.9	38.4
		4	3 par. Exponential	223.9	38.9	-4.1	-0.7	224	38.9
		5	2 par. Exponential	228.8	39.8	-6.9	-1.2	228.8	39.8
		-	Guiana Shield	221.1	38.4	5	0.9	221.1	38.4
		-	Pantropical	290.9	50.5	-95.2	-16.5	275	47.8
		-	Without height	278.3	48.4	-15.8	-2.7	278	48.3
Várzea	≥5 - <30	1	Quadratic	50.7	36	1.6	1.1	50.7	36.1
		3	Michaelis-Menten	50.9	36.2	2.5	1.8	50.9	36.2
		2	Weibull	50.7	36.1	1.2	0.8	50.7	36.1
		5	3 par. Exponential	50.9	36.2	0.8	0.6	50.9	36.2
		4	Log-linear	50.4	35.9	1.8	1.3	50.4	35.9
		-	Guiana Shield	94.5	67.2	42.5	30.2	84.5	60.1
		-	Pantropical	52.7	37.5	4.3	3.1	52.6	37.4
		-	Without height	56.8	40.4	-19.2	-13.7	53.5	38.1
	≥30	1	Quadratic	738.7	28.1	-59.7	-2.3	738.2	28.1
		3	Michaelis-Menten	746	28.4	-75.5	-2.9	744.1	28.3
		2	Weibull	738.6	28.1	-56.5	-2.1	738.3	28.1
		5	3. par. Exponential	746.7	28.4	-62.3	-2.4	746	28.4
		4	Log-linear	740.1	28.2	-53.5	-2	740.1	28.2

		-	Guiana Shield	1631.8	62.1	1076.5	41	1229.6	46.8
		-	Pantropical	1166.4	44.4	474.2	18	1068.4	40.6
		-	Without height	962.9	36.6	-429.7	-16.4	863.9	32.9
	≥5 (all trees)	1	Quadratic	402.8	53.5	-7.5	-1	403	53.5
		3	Michaelis-Menten	404.8	53.8	-10.7	-1.4	404.9	53.8
		2	Weibull	400.7	53.2	-7.2	-1	400.9	53.3
		5	3 par. Exponential	398	52.9	-9.4	-1.3	398.2	52.9
		4	Log-linear	416.3	55.3	-4.8	-0.6	416.5	55.3
		-	Guiana Shield	873.9	116.1	305.5	40.6	819.3	108.8
		-	Pantropical	680	90.3	131.3	17.4	667.6	88.7
		-	Without height	487.9	64.8	-115.5	-15.3	474.4	63
Both forests	≥1 - <30	3	Quadratic	64.5	43.9	3.2	2.2	64.4	43.9
		2	Michaelis-Menten	64.7	44.1	3.5	2.4	64.6	44
		1	Weibull	64.7	44.1	3	2	64.7	44.1
		4	3 par. Exponential	65.4	44.5	3.6	2.4	65.3	44.5
		5	2 par. Exponential	66.1	45	5.2	3.5	66	44.9
		-	Guiana Shield	75.9	51.7	16.8	11.4	74	50.4
		-	Pantropical	67.2	45.8	-16.4	-11.1	65.2	44.4
		-	Without height	63.9	43.5	-5.9	-4	63.6	43.3
	≥30	3	Quadratic	927	32.3	-20.9	-0.7	927.8	32.3

	2	Michaelis-Menten	927.6	32.3	-25.2	-0.9	928.3	32.4
	1	Weibull	921.6	32.1	-25.2	-0.9	922.3	32.2
	4	3 par. Exponential	922.9	32.2	-44.4	-1.5	922.9	32.2
	5	2 par. Exponential	931.8	32.5	-72.1	-2.5	930.1	32.4
	-	Guiana Shield	1175.5	41	495.2	17.3	1067.4	37.2
	-	Pantropical	1005.9	35.1	-63.1	-2.2	1005.1	35
	-	Without height	1115.9	38.9	169.3	5.9	1104.3	38.5
≥1 (all trees)	3	Quadratic	467.4	62	1.4	0.2	467.5	62
	2	Michaelis-Menten	468.4	62.1	0.7	0.1	468.5	62.1
	1	Weibull	461.1	61.1	0	0	461.3	61.1
	4	3 par. Exponential	457.6	60.7	-4.2	-0.6	457.7	60.7
	5	2 par. Exponential	459.6	60.9	-9.2	-1.2	459.6	60.9
	-	Guiana Shield	594.2	78.8	127.7	16.9	580.5	76.9
	-	Pantropical	526.8	69.8	-21.7	-2.9	526.4	69.8
	-	Without height	586.8	77.8	38.8	5.1	585.7	77.6
